# Anti-Melanoma Activities of Artemisone and Prenylated Amino-Artemisinins in Combination With Known Anticancer Drugs

**DOI:** 10.3389/fphar.2020.558894

**Published:** 2020-09-29

**Authors:** Ho Ning Wong, Angélique Lewies, Michaela Haigh, Joe M. Viljoen, Johannes F. Wentzel, Richard K. Haynes, Lissinda H. du Plessis

**Affiliations:** Centre of Excellence for Pharmaceutical Sciences (Pharmacen™), North-West University, Potchefstroom, South Africa

**Keywords:** artemisinin, artemisone, elesclomol, melanoma, redox dysregulation, prenylated piperazine-DHA derivatives

## Abstract

The most frequently occurring cancers are those of the skin, with melanoma being the leading cause of death due to skin cancer. Breakthroughs in chemotherapy have been achieved in certain cases, though only marginal advances have been made in treatment of metastatic melanoma. Strategies aimed at inducing redox dysregulation by use of reactive oxygen species (ROS) inducers present a promising approach to cancer chemotherapy. Here we use a rational combination of an oxidant drug combined with a redox or pro-oxidant drug to optimize the cytotoxic effect. Thus we demonstrate for the first time enhanced activity of the amino-artemisinin artemisone and novel prenylated piperazine derivatives derived from dihydroartemisinin as the oxidant component, and elesclomol-Cu(II) as the redox component, against human malignant melanoma cells A375 *in vitro*. The combinations caused a dose dependent decrease in cell numbers and increase in apoptosis. The results indicate that oxidant-redox drug combinations have considerable potential and warrant further investigation.

## Introduction

The body organ in which neoplasms occur most frequently is the skin, with over one million skin cancer cases recorded annually (Simões et al., 2015). Accounting for approximately 40% of all new cancer diagnoses, skin cancer represents a leading public health problem (Bray et al., 2018). Skin cancers are classified into two main groups based on the cell of origin and clinical behavior, firstly nonmelanoma skin cancers (NMSC) mainly originating in the keratinocytes, and secondly cutaneous malignant melanoma skin cancers, originating in the melanocytes (Simões et al., 2015; Gálvez et al., 2018; Que et al., 2018). Malignant melanoma is the most severe form of skin cancer, and are notably intractable to current treatment modalities; the average survival rate is 6–10 months after diagnosis. Furthermore, metastatic melanoma cells tend to disseminate to multiple organs including brain, bone, liver and lungs, rendering treatment strategies decidedly more challenging than in the case of NMSCs that often remain at the site of origin (Gálvez et al., 2018; Keung and Gershenwald, 2018). Surgery remains the standard treatment for melanoma where the bulk of the tumor must be excised. However, the peripheral part of the tumor cannot be eradicated completely and large areas of normal skin must also be excised (Que et al., 2018). Surgical resection of malignant melanoma that has metastasized must also be accompanied with complete lymph node dissection. However, there is a marked increase in risk of lymphedema due to a cascade of postoperative events (Jørgensen et al., 2018). Systemic adjuvant therapy, especially targeted combination therapy, might offer significant advantages in the treatment of melanoma.

Drastic modulation of intracellular oxidative stress generated through addition of specially selected xenobiotic agents has evolved as a strategy for inducing dysregulation in, and eventual destruction of, cancer cells (Cabello et al., 2009; Trachootham et al., 2009; Wondrak, 2009; Dharmaraja, 2017; Galadari et al., 2017; Lee et al., 2017). Oxidative stress is an imbalance between production of reactive oxygen species (ROS) that include superoxide, hydrogen peroxide, and hydroxyl radical, and the ability of the intracellular system to detoxify these species. Normally, ROS are generated at low steady state concentrations as by-products of cell metabolism, and are effectively neutralized by endogenous thiols such as reduced glutathione (GSH) generated from oxidized glutathione (GSSG) by NADPH-flavin disulfide reductases glutathione reductase (GR), thioredoxin reductase (TrxR) and others that maintain redox homeostasis in nonproliferating cells (Kirshner et al., 2008). However, the higher metabolic activity of proliferating cancer cells results in generation of elevated levels of ROS that requires enhanced turnover of NADPH-flavin disulfide reductases in order to maintain redox homeostasis. Thus, under these conditions, addition of any agent that acts to enhance ROS production will overwhelm the capacity of the antioxidant systems to control ROS. Normally, feedback results in enhanced production of NADPH *via* glucose-6-phosphate dehydrogenase (G6PD) in the hexose monophosphate shunt (HMS). Because G6PD is the rate-limiting enzyme, a “choke-point” is reached wherein demand for NADPH exceeds its supply. The excess ROS may then induce cell arrest and apoptosis (Kirshner et al., 2008; Cabello et al., 2009; Trachootham et al., 2009; Wondrak, 2009; Dharmaraja, 2017; Galadari et al., 2017; Lee et al., 2017). Overall, the enhanced oxidative stress due to uncontrollable generation of ROS affects the three major stages of cancer pathogenesis, namely proliferation, metastasis and development of resistance (Galadari et al., 2017).

**Figure 1:**
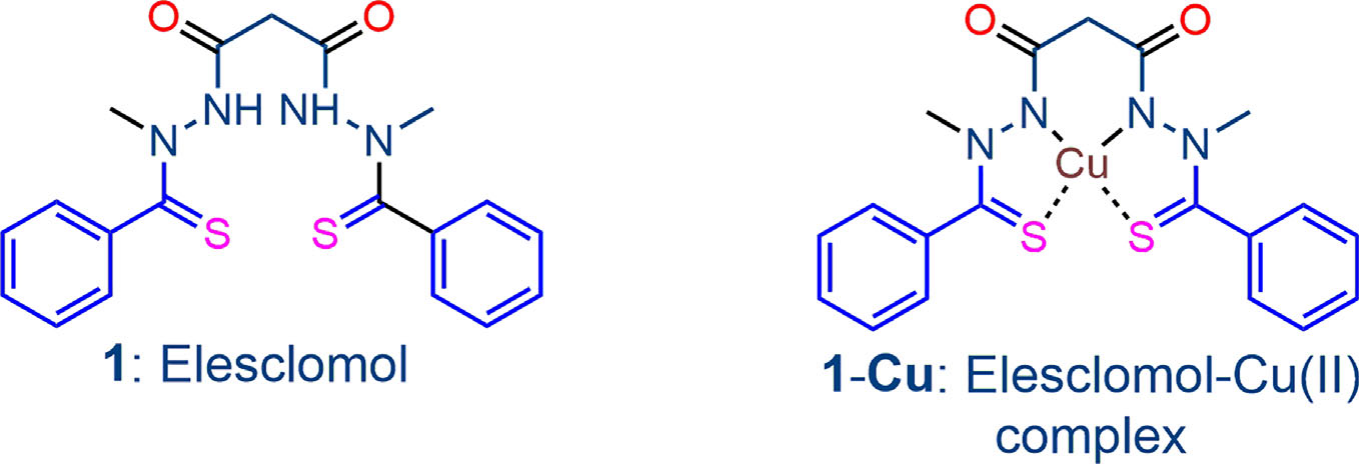
The bis-thionohydrazide elesclomol 1 and its stable square-planar copper(II) complex 1-Cu, the latter which is the redox-active drug.

Our strategy for inducing dysfunction in cancer cells also relies on the application of agents known to subvert redox homeostasis. However, here we use combinations that mutually amplify the effects of individual components. The use of redox (or “pro-oxidant”) drugs for this purpose is well-established (Cabello et al., 2009; Trachootham et al., 2009; Wondrak, 2009; Dharmaraja, 2017; Galadari et al., 2017; Lee et al., 2017). The redox drug is converted into its reduced conjugate that is reoxidized by atmospheric oxygen to restore the original redox drug and thereby generate ROS. The intracellular reductant that converts the redox drug into its reduced conjugate may be reduced flavin cofactors either associated with the NADPH-flavin disulfide reductases such as GR or TrxR important for maintaining redox homeostasis, or other flavoenzymes. The redox drug intercepts electrons *via* the reduced flavin cofactor from the flavin disulfide reductase, thus diverting electron supply required for generation of GSH from GSSG or other endogenous thiol. Thereby, with loss of homeostatic control, build-up of ROS involving intracellular redox processes now occurs. In addition, the redox cycling of the redox drug that ensues also provides a persistent, relatively high flux of ROS. The precept is well illustrated by the classic redox drug methylene blue (MB) that is rapidly reduced by reduced flavin adenine dinucleotide (FADH_2_) generated by NADPH-*E.coli* flavin reductase to leucomethylene blue (LMB)(Haynes et al., 2011). LMB in turn is rapidly oxidized by oxygen to MB with concomitant generation of ROS. MB also is rapidly reduced by NAD(P)H-flavin disulfide reductases such as TrxR, GR and lipoamide dehydrogenase (Buchholz et al., 2008a; Buchholz et al., 2008b; Buchholz et al., 2010) and thereby enhances oxidative stress both *indirectly* by diverting electron supply required for generation of antioxidant thiols from their disulfide precursors, and *directly* by oxidation of its reduced conjugate LMB by oxygen. Intracellular systems other than flavin disulfide reductases capable of providing electrons for converting redox drugs to their reduced conjugates also occurs. Thus, the highly potent antitumor agent deoxynyboquinone (DNQ) (Lee et al., 2017) generates superoxide *via* reduction by the cytosolic flavoenzyme NAD(P)H quinone oxidoreductase 1 (NQO1, DT-diaphorase that catalyzes metabolism of quinones) followed by oxidation by oxygen of the reduced DNQ intermediate. Although not demonstrated for DNQ, it is likely that the reduced flavin cofactor FADH_2_ of NQO1 rather than the primary electron donor NADPH directly reduces the DNQ as for other quinones (Sollner and Macheroux, 2009). Of relevance here is that reduced forms of redox active metal ions such as ferrous [Fe(II)] or cuprous [Cu(I)] ions also *directly* generate ROS through oxidation by oxygen. Thus, in principle, such redox active metal ions may serve as a surrogate of the redox drug – they have the same effect by generating ROS. However, in the presence of the metal ion, the immediate product of reduction of oxygen, namely superoxide, will be converted *via* hydrogen peroxide into hydroxyl radical by the Fenton reaction. In this respect, reduced flavin cofactors such as FADH_2_ are rapidly *oxidized* by transition metal ions such as Fe(III) that is thereby reduced to Fe(II); the reduction is considerably more facile than the established reduction of such metal ions by thiols such as GSH (Woodmansee and Imlay, 2002; Petrat et al., 2003). The precept is illustrated by the behavior of the bis-thionohydrazide elesclomol 1 (**Figure 1**), an anticancer drug which through sequestration of Cu(II) *in situ* is converted into the active elesclomol-Cu(II) chelate 1-Cu that generates ROS *via* redox cycling of the chelated Cu (Kirshner et al., 2008; Nagai et al., 2012). Apoptotic cell death in tumor cells follows from the enhanced oxidative stress induced by the complex (Blackman et al., 2012; Nagai et al., 2012). Elesclomol displays improved efficacy when used in combination with other drugs such as paclitaxel in human tumor xenograft models (Kirshner et al., 2008), and the combination has been used in a Phase III clinical trial in patients with melanoma, although progression free survival was not improved relative to controls (O’Day et al., 2013). Largely because of its proven mechanism of action and clinical use, and because of the possibility of establishing lower effective therapeutic doses through use in rational combinations, we initially use elesclomol-Cu (II) as the redox component in the current investigation.

**Figure 2:**
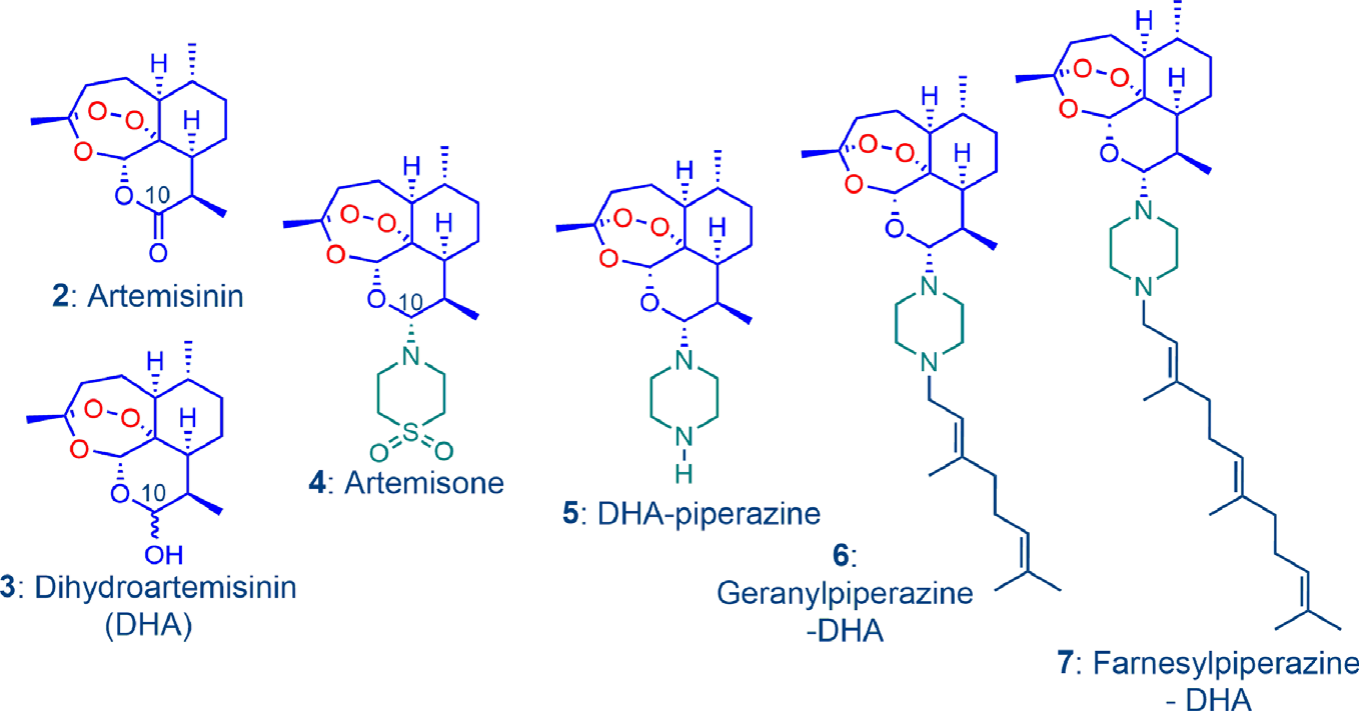
The amino-artemisinin derivative artemisone 4 is obtained from dihydroartemisinin (DHA) 3, that itself is a derivative of the peroxidic antimalarial drug artemisinin 2. Artemisone, and the novel geranylpiperazine-DHA 6 and farnesylpiperazine-DHA 7 derivatives prepared from DHA-piperazine 5 are the oxidant drug components of the combinations used in this work.

The oxidant drug component is differentiated from a redox drug through undergoing *irreversible* reduction to a reduced but usually inert form. While examples of such drugs that include aromatic amine *N*-oxides, nitro-imidazoles, quinones and others are given elsewhere (Wondrak, 2009), we focus here on derivatives of the antimalarial drug artemisinin 2. The second-generation derivative artemisone 4, prepared from dihydroartemisinin (DHA) 3, itself a derivative of artemisinin (Haynes et al., 2006; Chan et al., 2018) and the novel prenylated piperazine derivatives 6 and 7 prepared by direct alkylation by the prenyl bromide of the amino-artemisinin **5** bearing a piperazine group at C-10 (**Figure 2**) are included in this investigation. The compound 5 is readily obtained in one scalable step from DHA and piperazine (Wu et al., 2018; Wong et al., 2020). Of particular relevance to this work, it is noted that DHA 3 itself induces apoptosis in cultured human metastatic melanoma cells (A375, LOX and G361), with phosphatidylserine (PS) externalization, activation of procaspase 3 and increased generation of intracellular ROS (Cabello et al., 2012)

**Figure 3:**
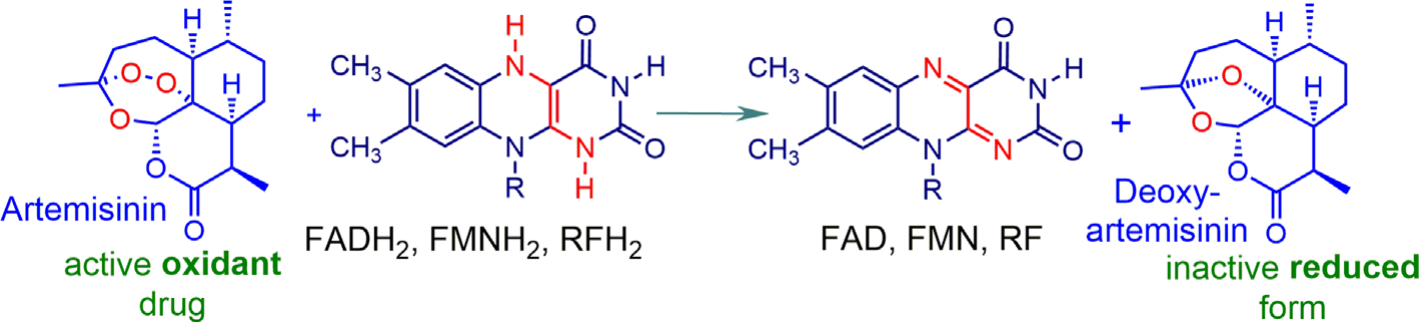
Artemisinin acting as an oxidant. Rapid oxidation of the reduced flavins of flavin adenine dinucleotide FAD, flavin mononucleotide FMN, riboflavin RF, and others takes place on exposure to artemisinin under physiological conditions. The products are the corresponding flavins and a ring opened form of deoxyartemisinin that undergoes ring closure to deoxyartemisinin (Haynes et al., 2010; Haynes et al., 2011; Haynes et al., 2012; Wu et al., 2016).

**Figure 4:**
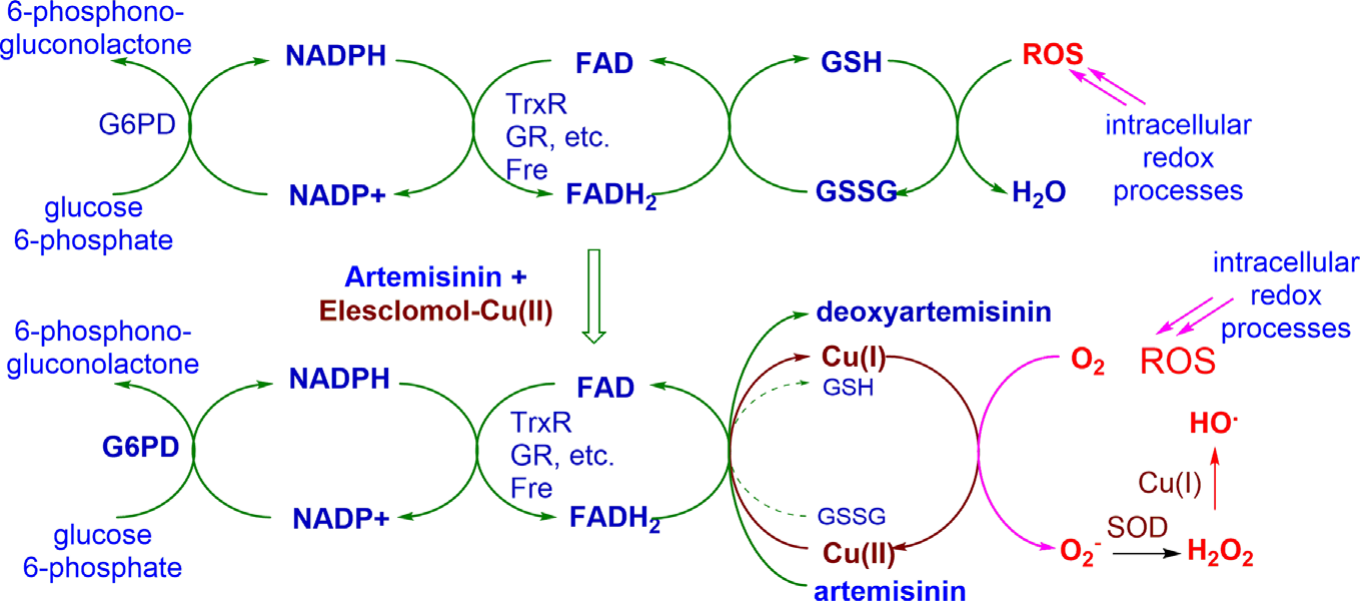
Conceptual basis for action of combination of oxidant drug artemisinin and redox drug elesclomol-Cu(II). Immediate ROS generation is triggered by the artemisinin, and is maintained by redox cycling of the metal ion derived from the redox drug. G6PD, glucose-6-phosphate dehydrogenase; NADPH, nicotinamide adenine dinucleotide phosphate; FAD, flavin adenine dinucleotide; TrxR, thioredoxin reductase; GR, glutathione reductase; Fre, flavin reductase; GSH, glutathione; GSSG, oxidized glutathione; ROS, reactive oxygen species; O_2_**-**, superoxide; SOD, superoxide dismutase; HO,. hydroxyl radical.

While the mechanism of action of artemisinins is a subject of considerable controversy, we have shown unequivocally that these rapidly oxidize reduced flavin cofactors such as FADH_2_ to the flavin (**Figure 3**). The reduced flavin in these mechanism studies is readily generated *via* use of NADPH-*E.coli* flavin reductase that mimics behavior of flavin disulfide reductases (Haynes et al., 2011). Thus, the oxidant drug artemisinin is itself incapable of directly generating ROS, but like the redox component, it intercepts electrons from reduced flavin cofactors of NAD(P)H-flavin disulfide reductases responsible for redox homeostasis, and other flavoenzymes (Trachootham et al., 2009; Haynes et al., 2010; Haynes et al., 2011). Thus, with the abrupt loss of homeostasis associated with an inability to generate thiols such as GSH (see above), rapid build-up of ROS ensues. However, as the oxidant artemisinin is irreversibly destroyed, re-establishment of redox homeostasis has to be suppressed so as to ensure prolongation of the cytotoxic effect. Thus, the redox drug must be used as a combination partner - under the combined effect of the two drugs, ROS build-up is now sustained. Although enzyme feedback results in enhanced production of NADPH *via* G6PD, the demand for NADPH exceeds its supply, and redox homeostasis is lost, resulting ultimately in cell death. The overall scheme is illustrated in **Figure 4** with artemisinin and the redox drug elesclomol-Cu(II).

Artemisone, like artemisinin and other antimalarial-active peroxides, also efficiently oxidize the reduced flavin cofactors (Haynes et al., 2011), but as an amino-artemisinin, is representative of a class of artemisinin that displays optimum biological activities on the basis of their oxidant mechanism of action (Wu et al., 2016). It exhibits both superior activity and safety in comparison to artemisinin resulting in enhanced antimalarial efficacy, improved bioavailability, metabolic stability, prolonged half-life and above all, absence of neurotoxicity (Haynes et al., 2006; Crespo-Ortiz and Wei, 2012). The compound also exhibits significant antitumor activity (Gravett et al., 2011; Crespo-Ortiz and Wei, 2012; Hooft van Huijsduijnen et al., 2013). Artemisone overall induces disruption of the cell cycle, resulting in growth arrest and hindering disease progression (Gravett et al., 2011; Crespo-Ortiz and Wei, 2012). Additionally, artemisone shows superior antitumor activity to artemisinin (Gravett et al., 2011). As noted below, when artemisone is formulated into monostearin solid lipid nanoparticles, the drug is selectively cytotoxic toward human melanoma A375 cells (Dwivedi et al., 2015).

**Figure 5:**
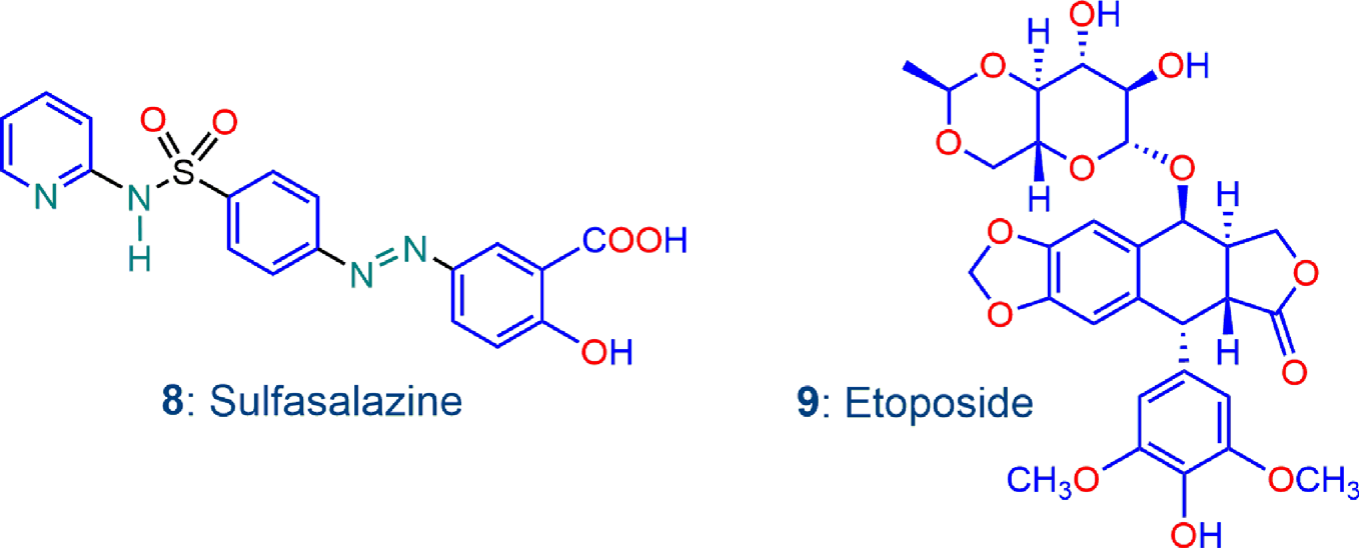
Additional compounds sulfasalazine 8 and etoposide 9 used in this work; both compounds are recorded to enhance oxidative stress.

We also briefly examine here the effects of adding other drugs to the artemisinin derivative that in one way or another are also known to enhance oxidative stress in a manner distinct to that involving the oxidant-redox drug pair combinations described above. Sulfasalazine 8 (**Figure 5**) is under investigation for its anticancer potential. It inhibits the cystine-glutamate antiporter xCT, a transmembrane protein that drives uptake of cystine in exchange for glutamate; with consequent intracellular reduction to cysteine, and conversion to GSH, xCT plays a critical upstream role in defense against oxidative stress. Thus, inhibition of xCT by sulfasalazine decreases cystine uptake and disrupts GSH production so as to sensitize cancer cells, in this case triple negative breast cancer cells, to treatment with anti-cancer agents that disrupt redox balance (Hasegawa et al., 2016). Likewise, sulfasalazine inhibits xCT in B16F10 melanoma cells, rendering them sensitive to treatment with hydrogen peroxide or to X-ray irradiation (Nagane et al., 2018). Etoposide 9 (**Figure 5**) exerts antitumor activity by inhibiting DNA topoisomerase II. In combination with bevacizumab, it is active against A375 melanoma cells (Calvani et al., 2016), but activity against acute myeloid leukemia cells in combination with the plant alkaloid homoharringtonine is linked to increased ROS generation and subversion of redox homeostasis. Interestingly it is etoposide that mediates ROS damage in the cell line; homoharringtonine alone does not generate ROS (Zhang et al., 2019). Thus examination of the effects of each of sulfasalazine and etoposide with our oxidant drugs is warranted.

Overall, the aim of this study is to investigate the anti-cancer efficacy of artemisone and aminoartemisinin derivatives toward melanoma cells A375, using nonmalignant keratinocytes (HaCat) as controls. The effects of combinations of the oxidant artemisinins with each of the redox active elesclomol-Cu(II), and the other drugs sulfasalazine and etoposide, on these cells would then be assessed. Cytotoxicity is assayed using the Sulforhodamine B and 3-(4,5-dimethylthiazol-2-yl)-2,5-diphenyl tetrazolium bromide (MTT) assays. The lactate dehydrogenase (LDH) assay is used to determine the effects of compounds on cell membrane integrity, and flow cytometry assays are used to assess apoptosis and necrosis end-points.

## Materials and Methods

### Materials

Artemisinin 2, m.p. 156°C–157°C, was obtained from the Kunming Pharmaceutical Corporation, Kunming, Yunnan Province, China, or from the Dang Quang Trading Company, Hanoi, Vietnam, and used as received. Artemisone 4 was prepared from dihydroartemisinin 3 and purified by recrystallization from isopropanol to give artemisone 4 as needles, m.p. 152-153°C (Haynes et al., 2006 152.3–152.7°C), as previously described; analysis by HPLC indicated a purity of ≥99% (Haynes et al., 2006; Chan et al., 2018). Elesclomol 1, 98% pure, was obtained from Kaixuan Chemical Company, Changzhou, Jiangsu, China, and used as such and as the elesclomol-Cu(II) complex 1-Cu. The complex was prepared *in situ* by treating the elesclomol with an equimolar amount of copper chloride dissolved in H_2_O (Yadav et al., 2013). Structure and purity of elesclomol were checked by means of ^1^H and ^13^C NMR spectroscopy; for the latter, the ^13^C NMR spectrum recorded in DMSO-d_6_ contained signals at δ 43.5 (CH_3_), 126 (Ar C-3),127 (Ar C-2), 128 (Ar C-4), 142 (Ar C-1),163 (C=O), 203 ppm (C=S), in agreement with literature data (Yadav et al., 2013). Sulfasalazine 8b, 97% pure, and etoposide 9, 98% pure, were purchased from Merck (Darmstadt, Germany) and used as received.

### Synthetic Chemistry

The full details of the reagents, instrumentation and the procedures used to convert DHA-piperazine 5 into the geranylpiperazine-DHA derivative 6 and the farnesylpiperazine-DHA derivative 7 (**Figure 2**) together with the full characterization data for the products are given in the **Supplemental Materials** and Methods - Chemistry Section.

### Cell Cultures

The human cell lines TK10 (human kidney adenocarcinoma), UACC62 (human melanoma) and MCF7 (human breast adenocarcinoma) were obtained from the National Cancer Institute (NCI) as part of a panel widely studied for drug screening and molecular target identification. Melanoma (A375) and human embryonic kidney (HEK293) were acquired from the American Type Culture Collection; ATCC^®^ CRL-1619™ and ATCC^®^ CRL-1573™ respectively. The nontumorigenic human immortalized keratinocyte (HaCaT) cells were a kind donation by the School of Anatomical Sciences, Faculty of Health Sciences, University of the Witwatersrand, South Africa. HaCaT cells were chosen as the nonmalignant control due to their ease of propagation, near normal phenotype and general use as an A375 control (Dwivedi et al., 2015; Lewies et al., 2018). The A375 cell line is a melanotic cells line with the BRAF mutation frequently used for *in vitro* drug screening assays for anti-melanoma drugs (Couto et al., 2019). All reagents were of analytical grade and were obtained from Merck (Darmstadt, Germany) unless stated otherwise. Cells were cultured in Dulbecco’s modified essential medium (DMEM; Hyclone, GE healthcare, South Logan, UT, USA) containing 10% fetal bovine serum (FBS), 1% penicillin/streptomycin, 1% 200 mM L-Glutamine and 1% nonessential amino acids (Lonza, Basel, Switzerland) at 37°C in a humidified atmosphere of 5% CO_2_. Stock solutions of the compounds were prepared in dimethyl sulfoxide (DMSO). All subsequent dilutions were prepared in serum-free DMEM and vehicle controls were included in all experiments

### *In Vitro* Cell Viability Assays

The growth inhibitory effects of the compounds were tested in the 3-cell line panel consisting of TK10 (renal), UACC62 (melanoma) and MCF7 (breast) cancer cells by Sulforhodamine B (SRB) assay. In addition, activity was also evaluated against HEK293 cells with the 3-(4,5-dimethylthiazol-2-yl)-2,5-diphenyl tetrazolium bromide (MTT) assay (Mosmann, 1983). Compounds prepared in DMSO were tested at 0.01–100 µM serial dilutions to establish initial inhibitory concentrations. The results were expressed as half maximal inhibitory concentrations (IC_50_) as described before (Lewies et al., 2018). After 24 h exposure to compounds, growth medium was removed, cells rinsed twice with 1 ml of phosphate buffered saline (PBS) and 100 µl fresh serum-free medium containing 5 mg/ml MTT solution was added MTT solution was prepared fresh for each analysis). Cells were then incubated for 4 h at 37°C, after which the MTT was removed and substituted with 100 µl dimethyl sulfoxide (DMSO) to dissolve the formazan crystals for an h at 37°C. After incubation, cell viability was determined using a microplate reader (BioTek^®^, Vermont, USA) at an excitation wavelength of 560 nm and emission wavelength of 630 nm with DMSO measured as a blank. Cell viability was expressed as a percentage relative to the untreated control, which is assumed to be 100% viable. For a positive control, cells were treated with 0.01% Triton-X 100 (Sigma-Aldrich, St Louis, MO, USA) for a period of 4 h. Using the MTT assay data, IC_50_ values were calculated using GraphPad Prism 8. Data were normalized to the positive (alleged to be 0% viable) and negative controls (presumed to be 100% viable), followed by the log-transformation of the concentration values. The curve was fitted using the log(inhibitor) vs. response function and the IC_50_ values calculated (Lewies et al., 2018). A crude therapeutic index (fold decrease in IC_50_ value) was calculated as the IC_50_ of the noncancerous cell-line/IC_50_ of the cancerous cell line. The Chou-Talalay experimental design of drug combinations (Chou and Talalay, 1984) were employed with drugs combined in a 1:1 ratio. Drug interactions were evaluated for synergism, additivity or antagonism using multiple drug effect analysis, based on the median-principle. Cells were treated with the drug combinations for 24 h and analyzed. The CompuSyn (ComboSyn, Inc. NJ, USA) software package was used to generate the median-effect plots and combination index plots. Based on the most effective compound combinations, membrane integrity assays and cytometric apoptosis and necrosis analyses were also performed. All experiments were performed at least in triplicate and repeated independently.

### LDH Assay

In order to determine cell membrane integrity, the CytoTox-ONE™ Homogeneous Membrane Integrity Assay (Promega, Madison, WO, USA) was employed. This fluorometric assay measures the release of LDH from cells with damaged cell membranes. Cells were seeded in a 96-well plate at a volume of 50 µl and incubated until cells were ~90% confluent as previously described (Malik et al., 1983; Wentzel et al., 2017). Cells were exposed to amounts of each compound and combinations corresponding to the respective IC_50_ values for 24 h. The manufacturer’s instructions were followed to determine the fluorescence at an excitation wavelength of 560 nm and an emission wavelength of 590 nm. In order to calculate the percentage cytotoxicity, the average fluorescent values of the background medium were subtracted from all average fluorescent values of the experimental samples and the positive control. Cytotoxicity is expressed as a percentage relative to the positive control, which is assumed to be responsible for 100% LDH release. The fold increase in LDH release was taken as the indication of the selectivity toward melanoma cell membranes, calculated as the % LDH release of the noncancerous cells/% LDH release of the cancerous cells. All experiments were performed at least in triplicate and independently repeated. Statistical significant differences were calculated with one-way ANOVA.

### Intracellular ROS Generation

HaCat and A375 cells were seeded at 25,000 cells/well in 96-well plates (Black plate, Clear bottom, Corning, Costar^®^, NY, USA) and incubated until cells were ~90% confluent. Serum-free DMEM without phenol red (Thermo Fisher Scientific, Gibco, Carlsbad, CA, USA) was used for all experiments. Cells were exposed to compounds or H_2_O_2_ (500 µM) for 12 h. Cell staining with ROS fluorescent indicator 2’,7’-dichlorodihydrofluorescein diacetate (DCFH-DA) was performed 1.5 h before the end of the 12-h exposure time. A kinetic analysis was then performed at 37°C using a microplate reader (SpectraMax^®^ ParadigmTM Molecular Devices, Sunnyvale, CA, USA) by measuring the DCF fluorescence (excitation 485 nm and emission 535 nm) every 10 min for 1 h. Following the analysis, the medium was removed and cells were gently rinsed twice with PBS. The Bradford assay (Coomassie blue colorimetric method) was then performed in order to determine the relative amount of protein per well, as an indication of cell number per well, and all fluorescence measurements were normalized relative to the cell number. The background fluorescence produced by blank medium controls and unstained but treated cell controls were subtracted from DCFH-DA stained cells. The DCF fluorescence measured at the beginning of the analysis (time 0) was subtracted from the DCF fluorescence at the indicated times for the kinetic analysis (results not shown). The intracellular ROS accumulation is also expressed as a fold change relative to the untreated control (set as 1) for the final measurement. All experiments were performed at least in triplicate and repeated independently.

### Flow Cytometry Detection of Apoptosis

The FITC Annexin V apoptosis detection kit I (BD Pharmigen™, BD Biosciences, San Jose, CA, USA) was used for the detection of apoptosis using flow cytometry. FITC Annexin V emits green fluorescence which indicates apoptosis, whereas propidium iodide (PI) emits red fluorescence and stains dead cells (Vermes et al., 1995). The assay was performed according to the manufacturer’s instructions with minor alterations. In brief, cells were seeded in a 96-well plate and grown to ~90% confluence. The cells were exposed to amounts of the prenylated piperazine derivatives and combinations with elesclomol-Cu(II) 1-Cu corresponding to the IC_50_ values. After 24 h, cells in each well were washed twice with 200 µl of PBS and resuspended in 1X binding buffer at a concentration of approximately 1 x 10^6^ cells/ml. The samples were then transferred to 12 × 75 mm flow tubes (BD Biosciences) and 5 µl of FITC Annexin V and 5 µl of PI was added. Samples were then briefly vortexed and incubated in the dark at room temperature for 20 min. After incubation, 100 µl 1X binding buffer was added to each tube and then analyzed using the BD FACSVerse™ and BD FACSuite™ Software. For a positive apoptosis control, cells were treated with 1 mM staurosporine (Sigma-Aldrich) for 4 h. At least 10,000 cellular events were analyzed for each sample. A logarithmic amplification scale was employed and events plotted on forward scatter (FSC), side scatter (SSC), green fluorescence (FL1) and red fluorescence (FL2). Gates were applied to these FSC/SSC dot plots to exclude cellular debris, multi-cell clusters and other background particles. Unstained negative controls, as well as positive controls, were included in all analyses. Flow cytometry procedure and gating strategies were described previously (Wentzel et al., 2017). Subsequent data analysis was performed using FCS Express 7 (De Novo Software, Pasadena, CA, USA). All experiments were performed at least in triplicate and independently repeated.

## Results

### Efficacies of Amino-Artemisinins Against Cancer Cells

The *in vitro* efficacies of the new derivatives were determined against various cancer cell lines, and expressed as IC_50_ values (**Table 1**). Artemisinin 2 was included in this study as a control compound. This is the parent compound from which all the derivatives are obtained; it has been shown to have anti-cancer properties (Buommino et al., 2009; Crespo-Ortiz and Wei, 2012). Artemisone 4 and farnesylpiperazine-DHA 7 were the most active compounds against the breast cancer (MCF-7) cell line. However, the latter derivative displayed potent activities against both the melanoma (UACC63) and kidney cancer (TK 10 and HEK293) cells as well.

**Table 1:** Cytotoxicities of the artemisinins against cancer cell lines *in vitro*.^ab^

Compound	HEK293	MCF-7	UACC62	TK 10
	IC_50_ µM			
Artemisinin 2	120.70 ± 1.07	ND	ND	ND
Artemisone 4	86.72 ± 1.09	2.75 ± 0.79	32.7 ± 3.47	>100
Geranylpiperazine-DHA 6	24.26 ± 1.15	ND	ND	ND
Farnesylpiperazine-DHA 7	35.46 ± 1.09	1.56 ± 0.51	2.15 ± 0.79	3.00 ± 0.67

a HEK293 human embryonic kidney; MCF-7 breast; UACC62 melanoma; TK10 renal cancer cell lines.

b Results are reported as inhibitory concentrations IC_50_ from three independent biological replicates, each performed as technical replicates ± standard deviation (SD).

**Figure 6:**
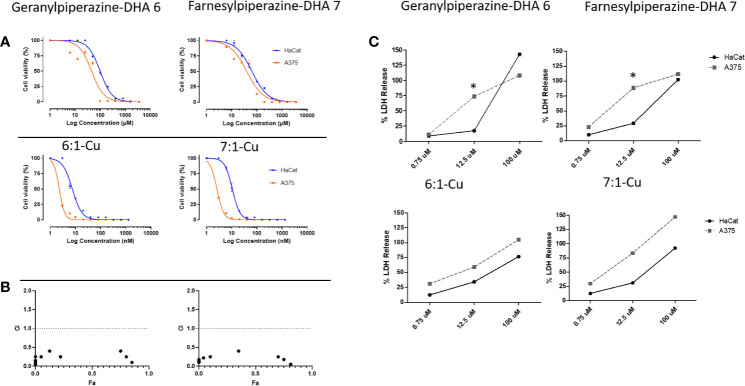
Efficacies of amino-artemisinins against normal keratinocytes (HaCat) and melanoma cells (A375). **(A)** Dose-response curves of geranylpiperazine-DHA 6 and farnesylpiperazine-DHA 7 (top panel) and their respective combinations with elesclomol-Cu(II) 1-Cu (bottom panel) determined with the MTT assay. **(B)** The fraction effected (Fa) vs the combination index (CI) plot for drug combinations at constant drug ratios. The dashed line indicates an additive effect, data points below 1 indicates synergism. **(C)** The effect of different concentrations of geranylpiperazine-DHA 6 and farnesylpiperazine-DHA 7 and their combinations with elesclomol-Cu(II) 1-Cu on membrane integrity determined with the LDH assay. Results are presented as mean ± SD, n=3. *Denotes statistical difference from the control.

Next, the quantitative colorimetric MTT assay was used to determine *in vitro* cell viability with A375 (melanoma) and HaCat (nonmalignant keratinocyte) cells. These were exposed to the artemisinin derivatives and combinations of these compounds with each of elesclomol-Cu(II) 1-Cu, sulfasalazine 8 and etoposide 9 for 24 h. In viable cells, NAD(P)H-dependent cellular oxidoreductase enzymes reduce MTT to formazan, and thus the amount of formazan produced reflects the number of viable cells present (Mosmann, 1983). The IC_50_ values of both malignant and nonmalignant cells and are summarized in **Table 2**. Artemisinin 2, artemisone 4, elesclomol-Cu(II) 1-Cu and etoposide 9 were active against the A375 melanoma cells with IC_50_ values comparable to those of obtained from other studies (Buommino et al., 2009; Blackman et al., 2012; Dwivedi et al., 2015; Calvani et al., 2016). The IC_50_ was used to determine a crude therapeutic index (fold increase or decline in IC_50_). Artemisinin 2 was 2.5 fold more active toward melanoma than toward nonproliferating cells and artemisone 4 and elesclomol-Cu (II) 1-Cu, were 2.8 fold more active toward the melanoma cells (**Table 2**). The anticancer drug etoposide 9 showed exceptionally good selectivity toward melanoma cells (therapeutic index of >297). Sulfasalazine 8 was the least active but was still selectively active against melanoma cells. These results are consistent with those of Nagane et al. (Nagane et al., 2018). The dose-response curves of the amino-artemisinins and the amino-artemisinins in combination with elesclomol-Cu 1-Cu are illustrated in **Figure 6A**. Geranylpiperazine-DHA 6 was more active against melanoma cells (IC_50_ of 46.94 µM) compared to nonmalignant cells (IC_50_ of 105.57 µM), and was more active than each of artemisinin 2 and artemisone 4. The combination of geranylpiperazine-DHA 6 with each of artemisinin 2, artemisone 4, etoposide 9 (**Table 2**) and elesclomol-Cu (II) 1-Cu (**Figure 6A**) also proved to be more selective toward melanoma cells than nonmalignant keratinocytes. Of special note is the combination of geranylpiperazine-DHA 6 with elesclomol-Cu(II) 1-Cu that led to a >255 fold increase in toxicity toward melanoma cells based on the IC_50_ values. Farnesylpiperazine-DHA 7 proved the most effective of all the single compounds tested in this study with at IC_50_ of 32.62 µM against melanoma cells. When farnesylpiperazine-DHA 7 was combined with these compounds, a reduction in the IC_50_ values against melanoma cells was generally observed (**Figure 6A**). The elesclomol-Cu(II) 1-Cu combination again proved to be the most selective toward melanoma cells, resulting in a >371 fold increase in toxicity.

**Table 2:** Cytotoxicities of the compounds and 1:1 combinations against melanoma (A375) and normal keratinocytes (HaCat).^a^

Compounds and 1:1 Combinations	A375 IC_50_ µM or nM^b^	HaCat IC_50_ µM or nM^b^	TI^c^	CI^d^
Artemisinin 2	149.7 ± 3.61	381.3 ± 5.36	2.5	
Artemisone 4	95.73 ± 2.31	268.8 ± 1.97	2.8	
Elesclomol-Cu(II) 1-Cu	1.72 ± 0.23 nM	4.88 ± 0.56 nM	2.8	
Sulfasalazine 8	569.80 ± 2.95	2223 ± 5.37	3.9	
Etoposide 9	43.66 ± 1.37	>12970	>297	
Artemisone 4 + Elesclomol 1-Cu	2.40 ± 0.47 nM	8.04 ± 0.49 nM	3.4	0.30
Geranylpiperazine-DHA 6	46.94 ± 0.89	105.57 ± 3.99	2.2	
6 + Elesclomol 1-Cu	<0.01 nM	2.55 ± 0.41 nM	>255	0.20
6 + Sulfasalazine 8	30.77 ± 0.27	134.3 ± 1.23	4.3	1.60
6 + Etoposide 9	20.88 ± 0.84	102.23 ± 2.67	4.8	0.74
Farnesylpiperazine-DHA 7	32.62 ± 1.08	70.64 ± 1.07	2.1	
7 + Elesclomol 1-Cu	<0.01 nM	3.71 ± 0.12 nM	>371	0.16
7 + Sulfasalazine 8	30.43 ± 1.14	61.43 ± 1.12	2	0.92
7 + Etoposide 9	10.87 ± 0.91	48.91 ± 1.01	4.5	0.42

a Results are reported as inhibitory concentrations IC_50_ in µM or nM from three independent biological replicates, each performed as technical replicates ± standard deviation (SD).

b Results from elesclomol-Cu(II) are reported in nM.

c Therapeutic index (TI) calculated as the fold decrease in IC_50_ of HaCat cells/IC_50_ of A375 cells.

d Combination index (CI): CI<1=synergism; CI=1 additive effect; CI>1=antagonism(Chou and Talalay, 1984; Chou, 2010). The drug interaction is more pronounced the farther a CI value is from 1.

### Synergism Analysis

In order to establish if combining the various compound types described here would result in lower effective therapeutic doses, we determined the efficacy of fixed dose combinations of various compounds according to the Chou-Talalay method (Chou and Talalay, 1984). The results are presented in **Figure 6B** and **Table 2** as the combination indices (CI). This method provides a quantitative assessment of synergism between two or more compounds. A value of CI less than 1 indicates synergism; while CI>1 indicates antagonism (Chou and Talalay, 1984; Chou, 2010). Compound effects are more prominent the farther a CI value is from 1. The geranylpiperazine 6 and farnesylpiperazine 7 derivatives were combined with other compounds in a 1:1 ratio. The CI was calculated at the IC_50_ value (also referred as the fraction affected or *F_a_* of 0.5) (Chou and Talalay, 1984; Chou, 2010). Artemisone and elesclomol-Cu(II) proved synergistic with a combination index of 0.30, well below 1. Geranylpiperazine-DHA 6 and farnesylpiperazine-DHA 7 and combinations with artemisinin, artemisone and sulfasalazine proved to be antagonistic or additive at the 50% *F_a_* concentrations (**Supplementary Figures S1, S2**). However, the combination of etoposide 9 with elesclomol-Cu(II) indicated synergistic interactions against melanoma cells. Notably, the combination of farnesylpiperazine-DHA 7 with elesclomol-Cu(II) elicited the most prominent synergism, as reflected in the very low CI value of 0.2.

### Cell Membrane Integrity Assay

In order to determine whether cytotoxicity is caused by physical cellular membrane damage, the LDH assay (Malik et al., 1983) was employed. Results are expressed relative to the untreated control and the maximum release sample, which was set as having 0% and 100% LDH release respectively. The LDH released from the nonmalignant HaCat cells and the A375 melanoma cells was then used to deduce the selectivity of the compounds against melanoma cell membranes. Exposure to each of artemisinin 2, artemisone 4, sulfasalazine 8, etoposide 9 and elesclomol-Cu (II) 1-Cu did not significantly increase the release of LDH (**Table 3**). On the other hand, exposure of the melanoma cells to the prenylated piperazine-DHA derivatives dramatically increased the LDH concentrations, indicating significant membrane damage (**Figure 6C**). Interestingly, none of the combinations with the prenylated piperazine-DHA derivatives had such a pronounced effect on LDH release as these derivatives on their own. This is an indication that the synergism of these compounds observed with each of etoposide 8 and elesclomol-Cu(II) is likely not attributable to membrane interaction or damage.

**Table 3:** Membrane integrity of normal keratinocyte HaCat and melanoma A375 cells after exposure to artemisinin derivatives.^a^

Compounds and Combinations^b^	HaCat(% LDH release)	A375 (% LDH release)	Fold increase in LDH release
Artemisinin 2	18 ± 0.84	24 ± 0.96	1.3
Artemisone 4	19 ± 0.79	27 ± 0.81	1.4
Elesclomol-Cu(II) 1-Cu	16 ± 0.79	21 ± 0.94	1.3
Sulfasalazine 8	35 ± 1.02	21 ± 0.96	0.6
Etoposide 9	7 ± 0.31	11 ± 0.56	1.5
Artemisone 4 + Elesclomol 1-Cu	20 ± 0.87	28 ± 0.87	1.4
Geranylpiperazine-DHA 6	17 ± 0.95	68 ± 0.91	4.0
6 +Elesclomol 1-Cu	24 ± 0.67	61 ± 1.12	2.5
6 +Sulfasalazine 8	59 ± 1.03	55 ± 1.03	0.9
6 + Etoposide 9	26 ± 0.56	64 ± 1.12	2.4
Farnesylpiperazine-DHA 7	18 ± 0.67	57 ± 1.21	3.1
7 +Elesclomol 1-Cu	31 ± 0.37	83 ± 1.24	2.6
7 +Sulfasalazine 8	48 ± 0.50	49 ± 0.64	1.0
7 + Etoposide 9	31 ± 0.65	26 ± 0.61	0.8

a Results are reported as %LDH release from three independent biological replicates, each performed as technical replicates ± standard deviation (SD).

b Compounds were administered at their IC_50_ concentrations. All combinations were administered in a 1:1 ratio.

### Detection of Intracellular ROS

**Figure 7:**
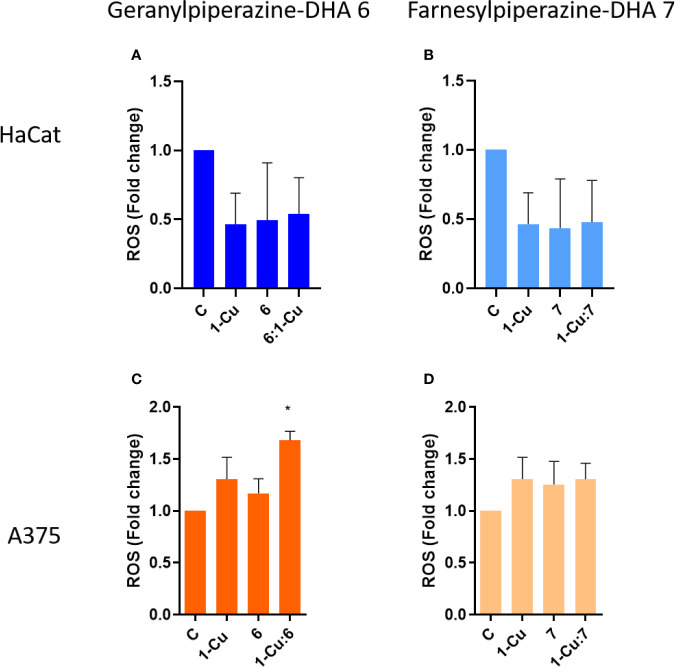
The intracellular reactive oxygen species (ROS) determined with the DCFH-DA assay, presented as DCF fluorescence fold change compared to the untreated control. **(A)** Normal keratinocytes (HaCat) cells and **(C)** melanoma (A375) cells treated with geranylpiperazine-DHA 6, elesclomol-Cu(II) 1-Cu and the combination 6:1-Cu. **(B)** Normal keratinocytes (HaCat) cells and **(D)** melanoma (A375) cells treated with farnesylpiperazine-DHA 7, elesclomol-Cu(II) 1-Cu and the combination 7:1-Cu. *Notes statistical significant difference when compared to the control.

The levels of intracellular ROS were determined using the DCFH-DA assay. This assay provides a measure of ROS as a global metric in conjunction with other efficacy screenings (Aranda et al., 2013; Nault et al., 2016; Ribou, 2016). Over a 12-h treatment period with the aminoartemisinins, DCF fluorescence did not markedly increase compared to the untreated control in the HaCat cells (**Figures 7A, B****)**. Treatment with elesclomol-Cu (II) 1-Cu also resulted in no significant increased intracellular ROS in HaCat cells. Compared to the normal keratinocytes, geranylpiperazine-DHA 6 induced formation of higher levels of intracellular ROS (**Figure 7C**). However, these were not significantly higher when compared to the untreated control. Geranylpiperazine-DHA 6 in combination with elesclomol-Cu(II) 1-Cu induced statistically significantly higher ROS levels when compared to the control. The levels of ROS induced by farnesylpiperazine-DHA 7 alone and in combination with 1-Cu was also higher in the melanoma cells, when compared to the normal keratinocytes, but not significantly higher than the untreated control (**Figure 7D**).

### Detection of Apoptosis and Late Apoptosis/Necrosis With Flow Cytometry

Flow cytometry was employed to establish if the observed decrease in cell viability in melanoma cells was of necrotic or apoptotic origin. Cultured melanoma and keratinocyte cells were exposed to predetermined IC_50_ concentrations of the prenylated piperazine-DHA derivatives and analyzed using the FITC Annexin V/PI flow cytometry assay. Annexin V is a Ca^2+^-dependent phospholipid binding protein with a high affinity for phosphatidylserine (PS). In early apoptosis this phospholipid is translocated from the outside of the plasma membrane. PS translocation precedes the loss of membrane integrity, a hallmark characteristic of late apoptosis or necrosis (Vermes et al., 1995). The representative flow cytometry dot-plots are illustrated in **Supplementary Figure 3**. For keratinocyte cells, the assay indicates that 84.1 ± 3.6% of the cells exposed to 45 µM geranylpiperazine-DHA 6 were still viable (FITC-Annexin V negative and PI negative). A slight decrease in cell viability was observed when the HaCat cells were exposed to farnesylpiperazine-DHA 7 (74.2 ± 2.6%). Exposure to combinations of the prenylated piperazine-DHA derivatives with elesclomol-Cu(II) did not result in drastic increase in apoptosis or necrosis and the majority of cells could still be considered viable (6 and 1-Cu 76.4 ± 3.9%, 7 and 1-Cu 70.2 ± 4.0%).

**Figure 8:**
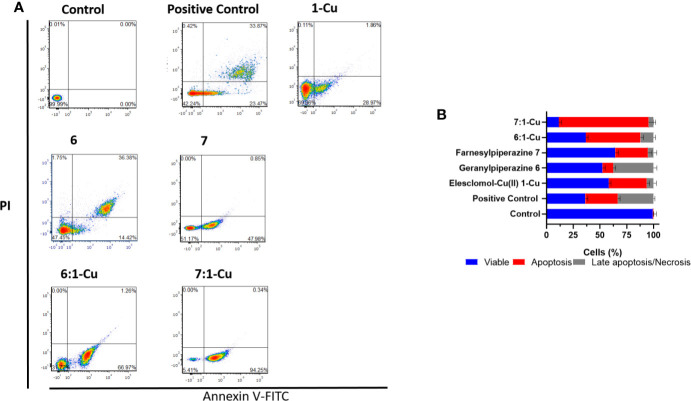
Apoptosis measured by FITC Annexin V/PI assay. **(A)** A375 cells were treated with elesclomol-Cu(II) 1-Cu (2 nM), geranylpiperazine-DHA 6 (45 µM) and farnesylpiperazine-DHA 7 (30 µM). Combinations in a 1:1 ratio were also included. Cells were double stained with Annexin V-FITC and PI for flow cytometry analysis and representative plots are shown. The flow cytometry plots are divided in the four quadrants with subpopulations of live (Annexin V and PI negative), early apoptotic (Annexin V positive and PI negative), late apoptotic (Annexin V and PI positive), and dead (Annexin V negative, PI positive) cells are indicated. **(B)** The bar graph illustrates the percentage cells of each grouping.

A considerably larger cell population (37.7 ± 2.1%) of melanoma cells exposed to 45 µM geranylpiperazine-DHA 6 underwent late apoptosis/necrosis (FITC-Annexin V positive and PI positive) than their keratinocyte counterparts (**Figures 8A, B**). A dramatic and significant increase in apoptosis (30.5 ± 3.7%) is observed in melanoma cells exposed to farnesylpiperazine-DHA 7. Exposure to the combination of 6 and 1-Cu resulted in a dramatic decrease in cell viability (36.6 ± 2.2% viable cells) with more than half of the cell population undergoing apoptosis (51.2 ± 2.8%). The combination of 7 and 1-Cu proved even more potent with 84.0 ± 4.3% of the melanoma cells undergoing apoptosis (**Figure 8B**). These results confirm the cytotoxicity data indicating that the prenylated piperazine-DHA derivatives are more selectively toxic toward melanoma cells. The combinations of the derivatives with 1-Cu showed significant increases in apoptosis compared to the individual treatments and this indicates that the cell death observed in these cells is most likely due to the activation of an apoptotic pathway/mechanism.

## Discussion

In this study, the efficacies of the novel aminoartemisinins geranyl- and farnesyl piperazine-DHA derivatives 6 and 7 in comparison with artemisinin 2 and artemisone 4 against cancer cells were evaluated. The farnesylpiperazine-DHA 8 is active against breast, kidney and melanoma cancer cell lines (**Table 1**). These results coupled with those from our previous study (Dwivedi et al., 2015), caused us to focus in more detail on melanoma cells. We thereby confirmed the anti-melanoma efficacies of artemisone 4 and elesclomol-Cu(II) 1-Cu against A375 melanoma cells. The IC_50_ values of the individual drugs (**Table 2**) are consistent with values obtained against other melanoma cell lines (Petrat et al., 2003; Kirshner et al., 2008; Hooft van Huijsduijnen et al., 2013; Dwivedi et al., 2015). For elesclomol-Cu(II) 1-Cu, the data confirm the necessity of copper for elesclomol to exert cytotoxicity (Nagai et al., 2012). The concentration of copper used in this study, 10 µM copper sulfate per 20 nM elesclomol, displayed similar toxicity relative to 10 µM copper chloride, used in other studies of elesclomol-Cu(II) (Yadav et al., 2013). Both artemisinin 2 and artemisone 4 demonstrated selectivity toward melanoma cells with therapeutic indices of 2.5 and 2.8 respectively. However, the anticancer drugs sulfasalazine 8 and etoposide 9 were more selective with etoposide having a therapeutic index of >297. These results are consistent with those of other studies involving these drugs on melanoma cells (Calvani et al., 2016; Nagane et al., 2018). The proposal to combine the oxidant artemisone 4 and the redox active elesclomol-Cu(II) 1-Cu to target proliferating cancer cells based on the susceptibility of cancer cells to oxidative stress was evaluated. The results (**Table 2**) indeed indicate the viability of the hypothesis of combining the oxidant drug (artemisone 4) with the redox-active drug elesclomol-Cu(II) 1-Cu (cf. **Figure 4**). In addition, strong synergism of the drugs at the concentrations tested was confirmed. The novel prenylated piperazine-DHA derivatives also displayed strong synergism with elesclomol-Cu(II) 1-Cu. Notably, the combinations of the aminoartemisinin derivative with etoposide also displayed synergism, and this important aspect will be further investigated elsewhere.

It was important to establish how the drugs induce cell death, as a prelude to understanding overall drug mechanism of action. Firstly, we establish if necrosis is the mode of cell death by using the LDH assay. The release of LDH from cells is a classic marker of plasma membrane damage (Malik et al., 1983), which is in turn a classic feature of necrosis. The progressive loss of plasma membrane integrity is also a feature of apoptosis described as secondary necrosis. It is hypothesized to occur *in vivo*, especially in tumor cells from patients undergoing chemotherapy (Zhang et al., 2018). Thus we find that artemisinin 2 and artemisone 4 did not induce significant LDH release. On the other hand, the prenylated piperazine-DHA derivatives 6 and 7 induced significantly higher levels of LDH release in melanoma cells with high selectivity toward the A375 cells compared to the HaCat cells. Whereas elesclomol-Cu(II) 1-Cu on its own did not induce LDH release, the combination thereof with both of the prenylated aminoartemisinin derivatives produced high levels of LDH release, with farnesylpiperazine-DHA 7 inducing 83 ± 1.24% LDH release. This finding cannot exclusively prove apoptosis *via* secondary necrosis as the mechanism of cell death, but it does give a clear indication of the extent of cell death after treatment with the compound.

The flipping of membrane phosphatidylserine (PS) is considered a classic feature of apoptosis, functioning as the “eat-me” signal to aid apoptotic cell recognition and clearance by phagocytes (Zhang et al., 2018). Both of the prenylated piperazine-DHA derivatives induced high levels of Annexin V positive staining as analyzed by flow cytometry and low levels of PI staining. This was indicative of PS flipping occurring after treatment with these compounds, relating to apoptosis as a mechanism of cell death occurring. When the compounds were combined with elesclomol-Cu(II) 1-Cu, the percentage apoptotic cells increased significantly with farnesylpiperazine-DHA 7 producing 84 ± 4.3% apoptotic cells. The derivatives were also selective toward melanoma cells, with higher percentages of apoptosis when compared to the HaCat cells. Levels of intracellular ROS were also increased in the melanoma cells relative to the HaCat cells. Additional studies to clarify the exact mechanism of action of these derivatives in combination with elesclomol-Cu(II) 1-Cu are planned.

In conclusion, redox directed combinations were investigated in which aminoartemisinins and elesclomol-Cu(II) 1-Cu were combined in order to potentiate activities against proliferating cancer cells based on the susceptibility of cancer cells to oxidative stress. The combination of artemisone 4 and elesclomol-Cu(II) 1-Cu was potently active against melanoma cells. In addition, we quantified the drug interaction between these two drugs and synergism was confirmed. The efficacy of novel prenylated piperazine-DHA derivatives 6 and 7 against melanoma cells *in vitro* was established, with the farnesylpiperazine-DHA 7 having a lower IC_50_ value. Combinations of the prenylated piperazine-DHA derivatives with sulfasalazine 8 and etoposide 9 did not significantly improve efficacy against melanoma cells. However, combinations of sulfasalazine 8 and etoposide 9 with elesclomol-Cu(II) 1-Cu significantly improved the selectivity and efficacy, with highly synergistic effects. Increased apoptosis in melanoma cells was demonstrated, but additional studies are needed to clarify the exact mechanism of apoptosis. Overall, the results described here indicate that the proposal for combining an oxidant drug with a redox-active drug appears to be successful in enhancing cytotoxicity against melanoma cells.

## Data Availability Statement

All datasets presented in this study are included in the article/**Supplementary Material**.

## Author Contributions

Inculcation of the concept and project overview was by RH. HW proposed additional structures and synthesized, characterized, and purified all the compounds. AL, MH, JV, and JW conducted the efficacy assays. JW and LP conducted the flow cytometry analysis. LP and RH wrote the draft manuscript. All authors contributed to the article and approved the submitted version.

## Funding

The South African National Research Foundation (NRF) is thanked for financial support to RH (NRF UIDs 90682 and 98934).

## Disclaimer

Any opinion, finding, and conclusion or recommendation expressed in this material is that of the authors, and the South African National Research Foundation does not accept any liability in this regard.

## Conflict of Interest

The authors declare that the research was conducted in the absence of any commercial or financial relationships that could be construed as a potential conflict of interest.
