# Supplementary material for: Anti-Melanoma Activities of Artemisone and Prenylated Amino-Artemisinins in Combination With Known Anticancer Drugs
Source: Front Pharmacol. 2020 Sep 29;11:558894. doi: 10.3389/fphar.2020.558894 (PMC7552967; doi:10.3389/fphar.2020.558894)
Supplement: Supplementary file 1 [file DataSheet_1.docx]

**Supplementary Data**

**Synthetic chemistry - *General***

The following solvents purchased from Sigma-Aldrich were AR grade and used as received: ethyl acetate, hexane, dichloromethane, toluene, diisopropylethylamine, triethylamine; tetrahydrofuran (THF) was HPLC grade and used as received. Geranyl bromide (95%) and *trans*,*trans*-farnesyl bromide (95%) were purchased from Sigma-Aldrich, and used as received. Thin layer chromatography was performed with Merck Kieselgel 60 F_254_ plates and visualized with ultra violet light (254 nm) or by placement in an iodine chamber. Column chromatography was performed with Merck silica gel 60 (0.04-0.063 mm). ^1^H NMR spectra were obtained on a 600 MHz Bruker Avance™ III spectrometers as solutions in CDCl_3_. ^1^H chemical shifts are reported downfield of tetramethylsilane (TMS), and were referenced to solvent signals in CDCl_3_ (7.26 ppm). Peak multiplicities are abbreviated as follows: s (singlet), d (doublet), dd (doublet of doublet), t (triplet), q (quartet) and m (multiplet). Coupling constants *J* are in Hz. NMR data were analyzed using MestReNova Software, version 5.3.2-4936. Infrared (IR) spectra were recorded on a Bruker Alpha-P FTIR instrument using the attenuated total reflectance technique. Mass spectral data were obtained on a Finnigan TSQ 7000 Mass Spectrometer operating in CI mode, and on a API QSTAR high performance triple quadrupole time-of-flight mass spectrometer with electrospray ionization. Infrared spectra were recorded on a Perkin Elmer Spectrum One spectrometer. Purity was assessed by HPLC analysis using an Agilent 1100 series instrument equipped with a gradient pump, autosampler, diode array UV detector and OpenLab CDS Chemstation Rev.C.01.07 SR3 data acquisition and analysis software (Agilent Technologies, Palo Alto, CA, USA).  The column was a Venusil XBP C18(2) -column, 150 x 4.6 mm, 5 µm spherical particles, 100 Å pore size (Agela Technologies, Newark, DE, USA). The mobile phase was acetonitrile and 0.1% orthophosphoric acid in water with a linear gradient from 30% acetonitrile to 85% after five minutes and holding until 15 minutes before equilibrating with 30% acetonitrile to 20 min. The flow rate was set at 1 mL/minute and the injection volume was 10 µL. The UV signal was monitored at 210 nm. For all samples, purity was ≥95%.

***DHA-Piperazine 5***

Dimethyl sulfoxide (DMSO) (25.1µL, 0.1 equiv) was added into a stirred solution of dihydroartemisinin (DHA) **3** (1 g, 3.5167 mmol) in toluene (10 mL) at room temperature under nitrogen in a 2-necked flask with a needle outlet. Oxalyl chloride (0.35 mL, 1.15 equiv.) was slowly added into the above reaction mixture and stirred for 1 h. The whole reaction mixture was transferred by a needle and syringe into a stirred solution of piperazine (1.5 g, 5 equiv.) in dichloromethane (10 mL) and stirred overnight. It was then quenched with saturated sodium bicarbonate solution (20 mL), extracted with ethyl acetate (4 x 30 mL), washed with brine (2 x 25 mL) dried with magnesium sulfate. After filtration and evaporation of solvent, the ratio of DHA-piperazine **5** to the by-product glycal was established by means of 1H NMR spectroscopy to be 2.1:1, corresponding to the presence of DHA piperazine 2.29 mmol, ca. 68% yield) according to the literature method (Wu et al., 2018; Wong et al., 2020). The crude material was used directly in the next step.

***Geranyl-piperazine-DHA 6***

The DHA-piperazine **5** (3.435 mmol) was dissolved in THF (30 mL) under nitrogen at room temperature. DIPEA (0.73 mL, 1.2 equiv.), followed by a geranyl bromide 95% (861.2 µL, 1.2 equiv.) were added into the above solution. The reaction mixture was stirred for 24 hr at room temperature. It was quenched by addition of saturated aqueous ammonium chloride solution (30 mL), and extracted with ethyl acetate (3 x 35 mL). The extracts were combined, washed with brine (40 mL) and dried over magnesium sulfate. After filtration, the filtrate was evaporated to dryness under reduced pressure, and the residue was submitted to chromatography. The product (322 mg, 19%) was isolated by elution with 30:70 ethyl acetate followed by 50:50 ethyl acetate:hexane as a colorless viscous oil. ^1^H NMR: δ = 0.79-0.82 (3H, d, J = 6.9 Hz, 9 Me), 0.93-0.95 (3H, d, J = 6 Hz, 6 Me), 1.35 (3H, s, 3 Me), 1.4-1.56 (3H, m), 1.59 (3H, s, Me group on the geranyl side chain), 1.63 (3H, s, Me group on the geranyl side chain), 1.67 (3H, s, Me group on the geranyl side chain), 1.70-1.75 (4H, m), 1.82-1.88 (1H, m), 1.97-2.10 (6H, m, proton from the geranyl side chain), 2.27-2.34 (1H, m), 2.38-2.54 (4H, m), 2.55-2.64 (1H, m), 2.67-2.72 (2H, m), 2.91-3.03 (4H, m, proton on the piperazine ring), 4.00-4.03 (1H, d, J = 10.2 Hz, H-10), 5.06-5.08 (1H, m, vinyl-H), 5.26 (1H, s, H-12), 5.26-5.30 (1H, m, vinyl-H). IR (film): v_max_ = 485.6, 510.1, 549.2, 2, 695.9, 744.7, 789.3, 812.3, 828.00, 852.0, 880.3, 894.6, 926.4, 943.0, 958.0, 983.0, 1012.7, 1055.5, 1085.7, 1105.5, 1119.1, 1131.0, 1161.7, 1178.9, 1206.5, 1240.2, 1278.4, 1292.9, 1326.7, 1376.2, 1453.1, 2810.4, 2871.9, 2927.2 cm^-1^. MS: *m/z*: calcd. for C_29_H_49_N_2_O_4_^+^ : 489.3692 [M+H]^+^; found : 489.3689.

***Farnesyl-piperazine-DHA 7***

The crude DHA-piperazine **5** (2.29 mmol) was dissolved in THF (20 mL) under nitrogen at room temperature. DIPEA (0.48 mL, 1.2 equiv), followed by farnesyl bromide (0.78 mL, 1.2 equiv) were added into the above solution. The reaction mixture was stirred for 22 hrs at room temperature. It was quenched by saturated ammonium chloride (NH_4_Cl) solution (20 mL), dilute with water (15 mL), then extracted with ethyl acetate (3 x 25 mL), washed with brine (30 mL) and dried with magnesium sulfate. The product (641 mg, 50%) was isolated by column chromatography (50% ethyl acetate in hexane with 1% triethylamine) as a colorless oil. ^1^H NMR: δ = 0.79-0.81 (3H, d, J = 7.2Hz, 9 Me), 0.93-0.95 (3H, d, J = 6 Hz, 6 Me), 1.35 (3H, s, 3 Me), 1.41-1.56 (3H, m), 1.59, 1.69 (6H, two singlet, overlapped, two Me groups on the farnesyl chain), 1.64 (3H, s, Me groups on the farnesyl side chain), 1.68 (3H, s, Me groups on the farnesyl side chain), 1.70-1.75 (4H, m), 1.82-1.88 (1H, m), 1.96-2.11 (10H, m, proton from the farnesyl side chain), 2.27-2.38 (1H, m), 2.39-2.54 (4H, m), 2.55-2.63 (1H, m), 2.66-2.72 (2H, m), 2.95-3.03 (4H, m, proton on the piperazine ring), 3.99-4.03 (1H, d, J = 10.2 Hz, H-10), 5.07-5.11 (2H, m, vinyl-H), 5.26 (1H, s, H-12), 5.28-5.31 (1H, m, vinyl-H). IR (film): v_max_ = 485.7, 509.7, 549.7, 617.8, 696.2, 744.8, 827.8, 851.5, 880.1, 894.5, 926.2, 942.7, 958.1, 982.6, 1012.8, 1041.5, 1055.6, 1085.5, 1105.9, 1130.8, 1161.4, 1178.9, 1206.0, 1227.3, 1240.0, 1278.5, 1292.6, 1326.5, 1376.2, 1451.3, 2871.7, 2926.0 cm^-1^. MS: *m/z*: calcd for C_34_H_57_N_2_O_4_^+^: 557.4318 [M+H]^+^; found : 577.4313.

**Drug combination studies**

We used the method of Chou-Talalay(Chou and Talalay, 1984) to determine drug interactions. Firstly, inhibition of proliferation in A375 (melanoma) cells was expressed as a percentage of the untreated control. Thereafter the log of the ratio [*f*a/*f*u] of the fraction of affected (*f*a) and unaffected (*f*u) was plotted against the log of the drug concentration. The resulting median effect lines allow for the calculation of the x-intercept (Log IC_50_) and the slope (m). The calculated parameters are used to calculate the doses of the individual compounds required to produce proliferation inhibition according to equation 1. For each level of inhibitory concentration, the combination index (CI) values were calculated according to equation 2. CI values of <1 indicate synergy, values>1 indicate antagonism and values equal to 1 indicate additive effects. The relative degree of the interaction was determined based on the value relative to 1; therefore, the smaller the value, the greater the degree of synergism.

Dose_1_ = Dose IC_50_ [1 - *f*)/*f*]^1/m^ [1]

CI = (*D*)_1_/(*D_x_*)_1_ + (*D*)_2_/(*D_x_*)_2_ + α (*D*)_1_(*D*)_2_/(*D_x_*)_1_(*D_x_*)_2_ [2]

(*D_x_*)_1_, dose of drug 1 required to produce *x%* effect alone, (D)_1_, dose of drug 1 to produce the same *x%* in combination with drug 2 (*D*)_2_. (*D_x_*)_2_, dose of drug 2 required to produce *x%* alone, (*D*)_2_, dose of drug 2 to produce the same effect in combination with (*D*)_1_.

| 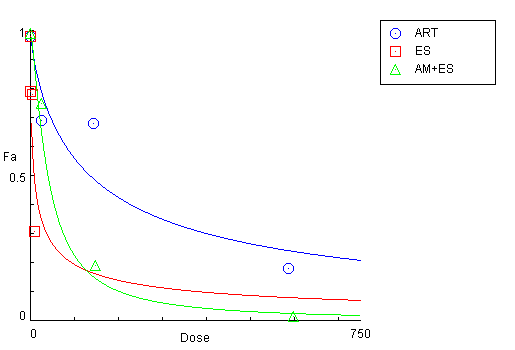  A | 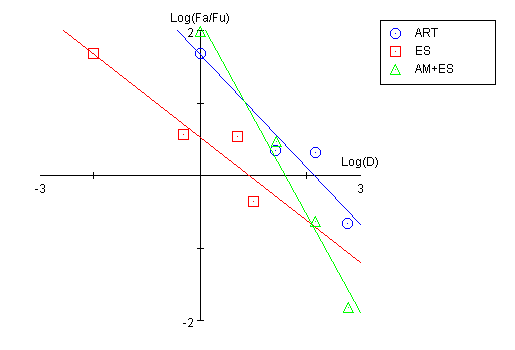  B |
| --- | --- |
| 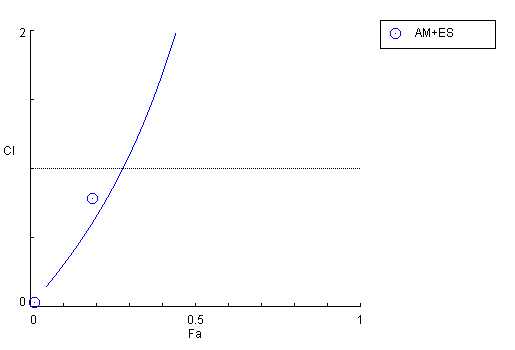  C | 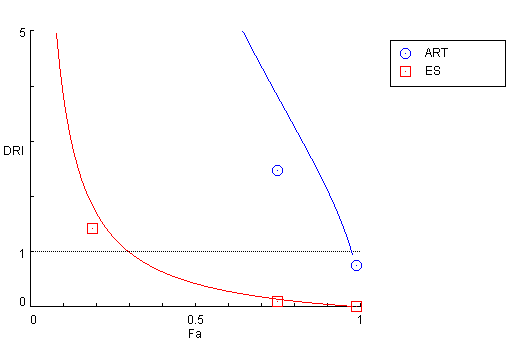  D |

**Supplementary Figure 1.** Drug interaction between artemisone and elesclomol-Cu(II) where A) Fa/dose plot to calculate the IC_50_ of artemisone (ART), elesclomol-Cu(II), (ES) and the combination of the two drugs, AM+ES. B) The log Fa and Log IC_50_ plot to calculate the dose, C) the Fa/CI plot to calculate the drug interaction, D) the Fa-DRI plot for the constant ration combination design.

The resulting quantitative diagnostic plots generated by computer simulation are illustrated in Figure S1. The combination of artemisone and elesclomol-Cu(II) is provided for illustrative purposes. It is important to note that the curve fitting of the software assumes linearity of the data as is illustrated in Figure S1 (B). If the data is not linear, the equation may overestimate antagonism due to incorrect interpolation from the curve. Rather than producing a true antagonistic interaction, an ambiguous fit is produced with large (>10) CI values (Chou, 2010).

| 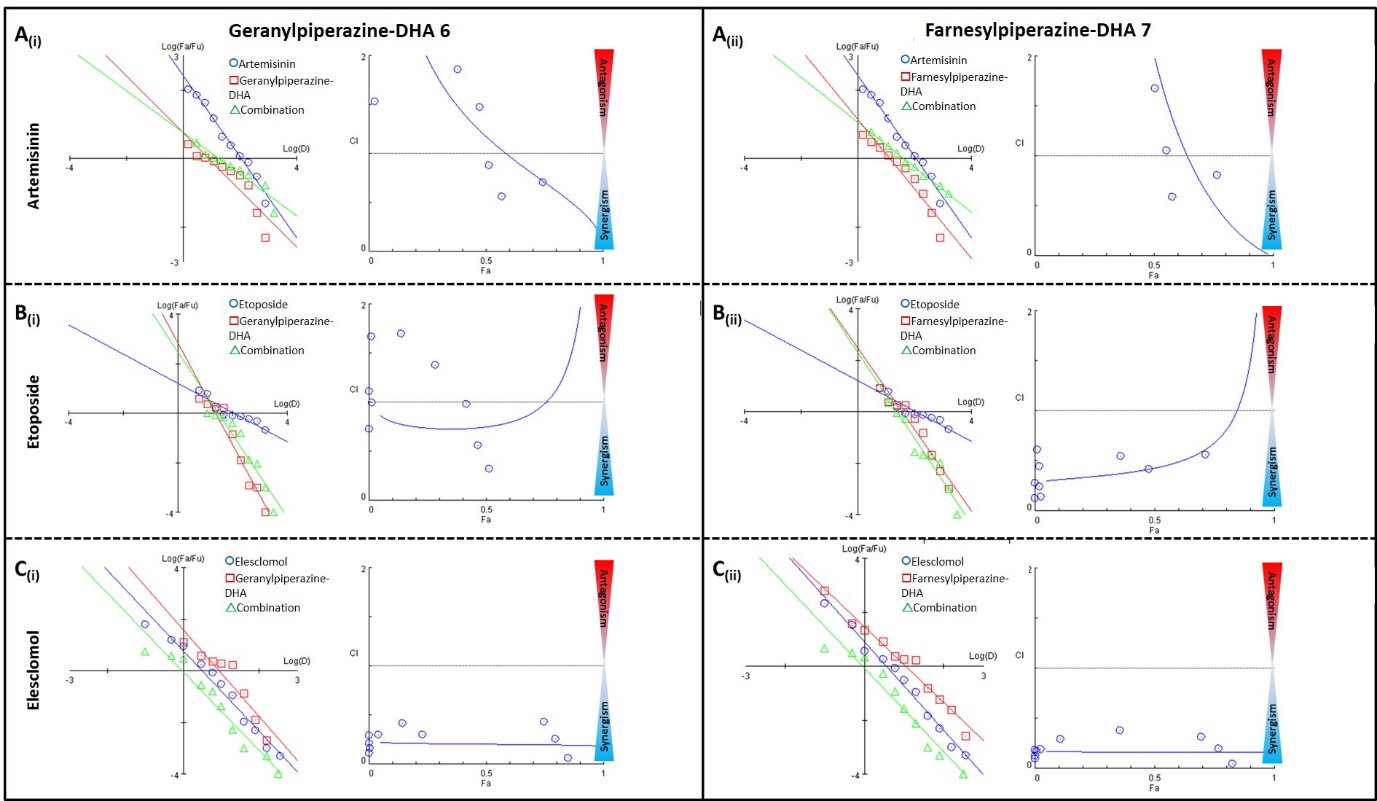 |
| --- |

**Supplementary Figure 2.** Exposure of A375 cells to artemisinin, etoposide and elesclomol-Cu(II) in combination with geranylpiperazine-DHA **6** and farnesylpiperazine-DHA **7** for 24 h in a fixed ratio combination of 1:1. A) the Log Fa/Fu or IC_50_ vs Log (D) plots. B) The fraction effected (Fa) vs the combination index (CI) plot for drug combinations at constant drug ratios. The dashed line indicates an additive effect, data points below 1 indicates synergism and data points above 1 indicates antagonism. The line indicate the computer simulated response and the closed symbols are the actual data points of the drug combination.

| 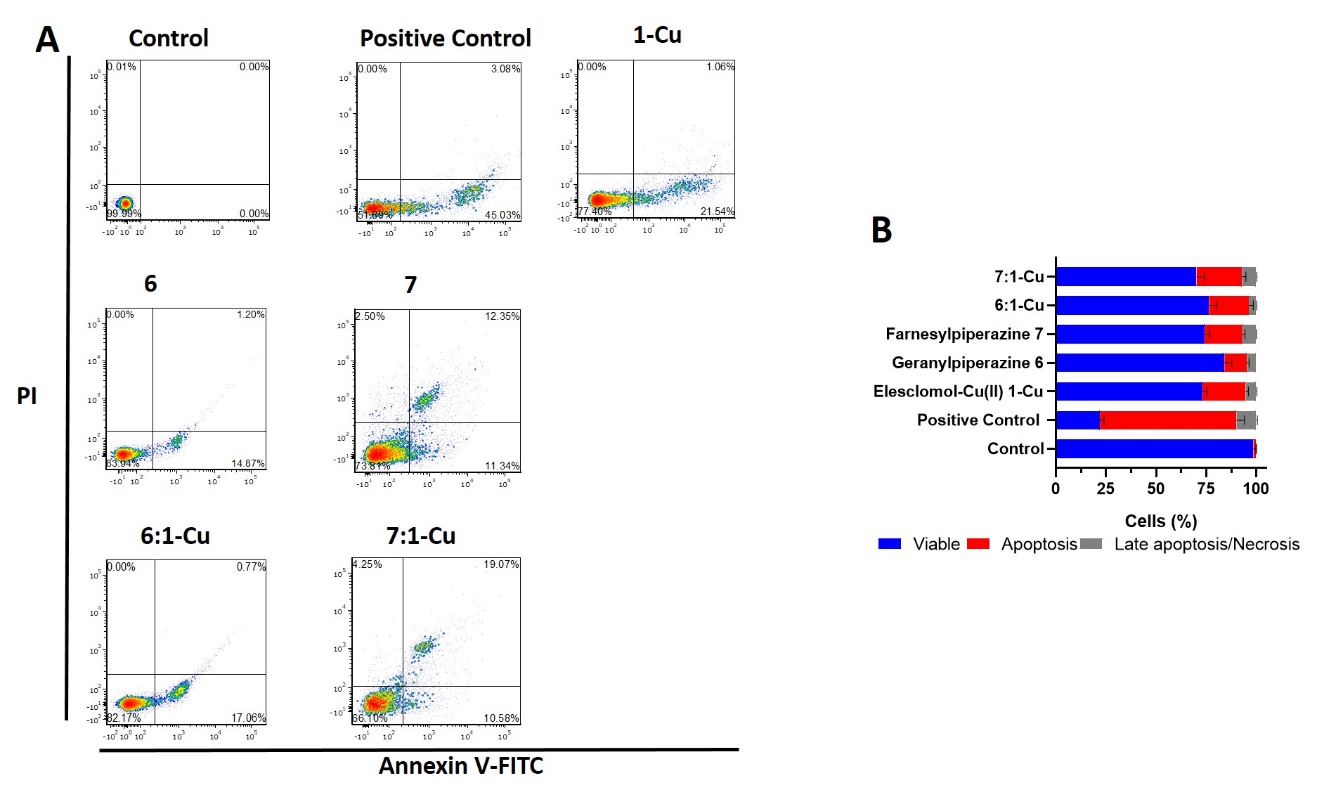 |
| --- |

**Supplementary Figure 3.** Apoptosis measured by FITC Annexin V / PI assay. A) HaCat cells were treated with elesclomol-Cu(II) **1-Cu** (2nM), geranylpiperazine-DHA **6** (45 µM) and farnesylpiperazine-DHA **7** (30 µM). Combinations in a 1:1 ratio were also used. Cells were double stained with Annexin V-FITC and PI for flow cytometry analysis and representative plots are shown. The flow cytometry plots are divided in the four quadrants with subpopulations of live (Annexin V and PI negative), early apoptotic (Annexin V positive and PI negative), late apoptotic (Annexin V and PI positive), and dead (Annexin V negative, PI positive) cells are indicated. B) The bar graph illustrates the percentage cells of each grouping.

References

Chou, T.C. (2010). Drug combination studies and their synergy quantification using the Chou-Talalay method. *Cancer Res* 70(2)**,** 440-446. doi: 10.1158/0008-5472.can-09-1947.

Chou, T.C., and Talalay, P. (1984). Quantitative analysis of dose-effect relationships: the combined effects of multiple drugs or enzyme inhibitors. *Adv Enzyme Regul* 22**,** 27-55. doi: 10.1016/0065-2571(84)90007-4.
